# Interrogating the Azo-Hydrazo Proton Transfer Process in the Excited State Using a Multipronged Spectroscopic Study: Effect of Annulation and Solvent

**DOI:** 10.1021/acsphyschemau.5c00067

**Published:** 2025-09-11

**Authors:** Jack Dalton, Vasilios G. Stavros, Arghyadeep Bhattacharyya

**Affiliations:** † School of Chemistry, 1724 University of Birmingham, Edgbaston, Birmingham B15 2TT, U.K.; ‡ Department of Chemistry, 72896 Tripura University (A Central University), Suryamaninagar, Agartala 799022, India

**Keywords:** synthesis, emission, tautomerism, azo-dyes, transient absorption

## Abstract

Excited state intramolecular proton transfer (ESIPT) in organic dyes is one of the most studied photophysical processes by experimental and theoretical chemists alike. Of the various subclasses of ESIPT, the azo-hydrazo tautomerism occurs in *ortho* azo-dyes derived from phenols, mainly β-naphthol. In the current study, we present a steady-state and time-resolved spectroscopic investigation on the azo-hydrazo proton transfer process in two azo dyes derived from β-naphthol, 1-phenylazo-2-naphthol (**PDNO**) and 1-naphthylazo-2-naphthol (**NDNO**), which differ by a phenyl ring in solvents of differing polarity and proticity. Steady-state investigations implicate the presence of the ground-state azo-hydrazo tautomerism. The emission lifetime measurements reveal that the azo and hydrazo tautomers have different radiative lifetimes, with the hydrazo tautomer decaying faster than the azo tautomer. The femtosecond-picosecond transient absorption measurements provide critical information on the nonradiative decay processes associated with the dyes and demarcate the temporal behavior of the nonradiative relaxation between the two dyes. **NDNO**, having an extra phenyl ring, reveals slower relaxation times compared to **PDNO**, irrespective of the solvent. The relaxation time is longest in chloroform and shortest for nonpolar hexane, irrespective of dye. Considering both the steady-state and time-resolved measurements, we propose how the spectrodynamics in azo dyes derived from β-naphthol could be manipulated by tuning the annulation as well as the surrounding solvent system, which aids the fundamental understanding of excited-state photoprocesses like the azo-hydrazo tautomerism.

## Graphical abstract

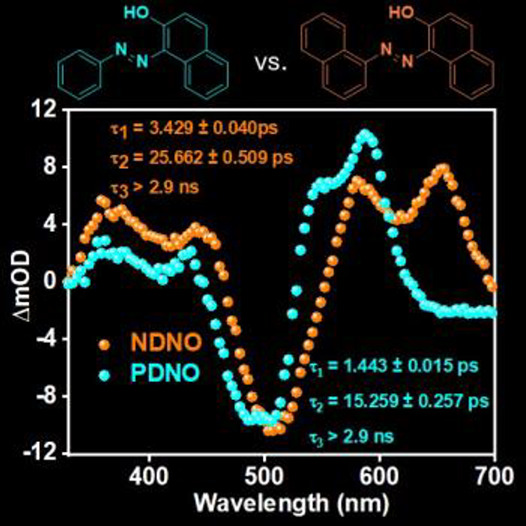

## Introduction

The systematic spectroscopic dissection of excited-state photoprocesses of organic dyes has been an area of mounting interest to spectroscopists, principally for a better understanding of how light-matter interaction affects the optoelectronic properties of the dyes.^1–8^ Among the various photoprocesses studied in organic dyes, the excited state intramolecular proton transfer (ESIPT) has been one of the most extensively studied, owing to the importance of proton transfer (PT) in various biological processes.^9–14^ A molecule undergoing PT, which leads to the formation of a tautomer in turn, can be categorized into the following pathways: (i) The tautomer is formed exclusively in the excited state, like that of methyl salicylate and 2-hydroxyophenylbenzazoles.^15–17^ This pathway is more commonly referred to as ESIPT. (ii) The molecule can undergo ESIPT in addition to ground-state intramolecular PT (GSIPT), whereby both tautomers exist in the ground state. This pathway occurs in some Schiff bases (imine-amine tautomerism) and azo dyes (azo-hydrazo tautomerism) derived from β-naphthol.^18–22^

Interestingly, although the pathway (i) has been extensively studied by an array of spectroscopic methods, pathway (ii) has received less attention. The case of the Schiff bases undergoing GSIPT has been reported as a special case (as PT has been observed to occur mainly in the excited state), whereas that of the azo dyes has been reported to be a general case with both tautomers present in the ground state due to the low energy barrier of tautomerism supported by theoretical calculations as well.^23,24^ The azo dyes are reported to be weakly emissive, thereby indicating a dominance of nonradiative decay pathways over radiative ones.^22^ Femtosecond-picosecond (fs-ps) transient absorption (TA) has been employed to observe the tautomerism process in the benchmark molecule 1-phenylazo-2-naphthol (**PDNO**; see Scheme 1 for structure), also known as Sudan-I. The studies reported involved the confinement of **PDNO** in various mesoporous materials in which their photodynamics were measured using both fs-TA and picosecond-nanosecond (ps-ns) time-resolved experiments,^19^ observing the isomerization dynamics of **PDNO** in cyclohexane-methylcyclohexane mixtures.^21^ These studies,^19^ while informative, still leave open questions. For example, the (i) effect of solvent polarity; (ii) effect of proticity; and (iii) effect of the introduction of an extra benzene ring to the **PDNO** moiety, all of which could potentially affect the isomerization process in **PDNO**.

**Scheme 1:**
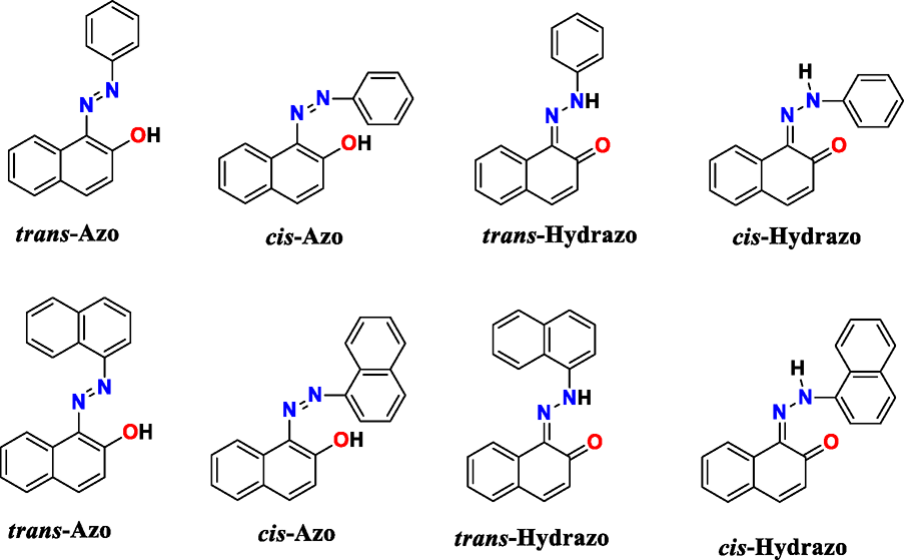
Various Tautomeric Forms of **PDNO** (Top Row) and **NDNO** (Bottom Row) That Shall Be Used in the Discussion of the Spectroscopy^a^

a The azo linkage refers to the −N=N connectivity whereas the hydrazo linkage corresponds to the =N–NH– connectivity.

Here, we present a multipronged study involving steady-state and time-resolved (ps-ns emission lifetime measurement and fs-TA measurements) spectroscopy of **PDNO** and its naphthalene analogue 1-naphthylazo-2-naphthol (**NDNO**) in a series of solvents of varying polarity, proticity, and viscosity. We started from the established results of the ultrafast nature of the azo-hydrazo tautomerism (τ_azo‑hydrazo_ < 2 ps)^19^ and the differential spectral signature of the azo and hydrazo tautomers observed by fs-ps transient absorption spectroscopy.^22^ In the current work, we focused on the spectral signature of the two dyes in an array of solvents in terms of their picosecond-to-nanosecond emission lifetime and femtosecond-to-picosecond TA studies. The steady-state studies reveal the presence of both the azo and hydrazo tautomers, and excitation at the absorption of each leads to emission, albeit extremely weakly, from the azo and hydrazo tautomers. The picosecond-to-nanosecond emission lifetime studies reveal the presence of multiple emissive species in the excited state owing to the presence of the two tautomers; our selective excitation of each tautomer leads to the disentangling of the radiative relaxation process of each. Our fs-TA studies show that the isomerization process becomes restricted in chloroform for both **PDNO** and **NDNO,** while the overall photodynamics are unchanged as we move from *n*-heptane to methanol. Interestingly, the introduction of an extra phenyl ring in **NDNO** leads to a considerable reduction in the relaxation rates compared to **PDNO**, as evidenced from the fs-TA experiments. Our presented experiments show the time constants of radiative rates of decays on the ps-ns scale. On the other hand, our fs-ps TA studies shed light on the nonradiative time constants of the tautomeric forms of the dyes post photoisomerization. The extracted time constants from the femtosecond-picosecond TA experiments showed a nice variation in their values upon change in the solvent as well as the increment in the annulation, as has been described in the manuscript. These two aspects indeed provided new aspects that were otherwise unattended in the previous studies.

In addition to these spectroscopic studies, we have also isolated the crystal structure of **NDNO,** where the presence of intramolecular hydrogen bonding was observed between the phenolic −OH group and the nitrogen atom of the diazo linkage existing in the dyes. It has been observed for molecules undergoing PT that the presence of such an intramolecular hydrogen-bonded ring between the proton donor (the −OH group in the current case) and the acceptor (the diazo linkage in the current case) acts as a crucial prerequisite for the possibility of a PT reaction, irrespective of its occurrence in the ground state or the excited state.^25^ As the intramolecular hydrogen bonding interaction is reported to be preserved in nonpolar to moderately polar solvents and ruptured in polar and protic solvents, this is a key step for explaining the isomerism as well as the apparent nonvariance of the spectrodynamic behavior of the dyes in solvents other than chloroform.

## Experimental Section

### Chemicals and Apparatus

β-naphthol, aniline, and 1-aminonaphthalene were purchased from Sigma-Aldrich and used without further purification. Spectroscopic grade solvents were used for the spectroscopic studies. The following abbreviations were used for the solvents: CHCl_3_ for chloroform, DMF for dimethylformamide, and MeOH for methanol. Due to unequal solubility of the dyes in the solvents used, the stock solutions of the dyes in each solvent were diluted in the respective solvents (*n*-heptane, CHCl_3_, DMF, and MeOH) to give equal values of absorption in each case, for proper comparison. The same approach was used prior to conducting the fs-TA experiments.

^1^H NMR and ^13^C NMR spectra were recorded on a Bruker 300 MHz spectrometer, using a CDCl_3_ solution with TMS as the internal standard.

The single crystal for **NDNO** was obtained by the slow evaporation of saturated solutions of the chalcones in chloroform over a period of a few days. The single crystals of **NDNO** were mounted on a Bruker-AXS SMART APEX II diffractometer equipped with a graphite monochromator and Mo Kα (λ = 0.71073 A) radiation. The crystal was placed 60 mm away from the CCD, and 360 frames were measured with a counting time of 10 s. The structure was solved using the Patterson method with SHELXS 97. Subsequent difference Fourier synthesis and least-squares refinement revealed the positions of the remaining non-hydrogen atoms. Non-hydrogen atoms were refined with independent anisotropic displacement parameters. Hydrogen atoms were placed in idealized positions, and their displacement parameters were fixed to be 1.2 times larger than those of the attached non-hydrogen atoms. Successful convergence was indicated by the maximum shift/error of 0.001 for the last cycle of the least-squares refinement.^25^

Absorption spectra were recorded on a Shimadzu UV-1900 absorption spectrophotometer. Emission spectra were collected by using a Shimadzu RF-6000 emission spectrophotometer. Emission lifetime decays were recorded on a Horiba Jobin Yvon FL3C-11 time-resolved emission spectrophotometer by exciting the samples using pulsed laser diodes of 451 and 502 nm, respectively. The instrument response function (IRF) for each laser source, estimated by observing the pulse width upon scattering using a very dilute sodium dodecyl sulfonate (SDS) solution, was typically ∼80–120 ps.

The emission lifetime decays were deconvoluted and analyzed using the DAS 6.0 package by Horiba, which allows local and global fits, and the instrument could successfully detect up to a ∼50 ps lifetime component. The emission decays were collected at the magic angle (54.7°) to exclude any effect of anisotropy. Since the emission of the dyes used was very poor, in that case, acquiring the decays until 10,000 counts often proves more detrimental to the overall signal and the deconvolution thereof, particularly for the decays collected at the blue end of the spectrum, where the incident laser may “sip-in” and the deconvolution of such a signal may furnish abnormally short lifetime components. For this reason, we collected all signals until 5000, which is neither too low nor too high, and maintained parity in all the measurements. Another support for the reliability of our presented data is the observation of the signals and the “second pulse” of the laser source in the IRF. Even the fastest of decays presented in the paper and the Supporting Information were devoid of the second pulse, which attests to the good signal-to-noise ratio of the presented decays.

The fs-TA setup has been outlined in detail previously, and only details specific to this experiment are presented here.^26^ The pump wavelength was set to 480 nm for **PDNO** and 505 nm for **NDNO** (near the maximum absorbance for each), and the pump pulse energy was set to ∼500 nJ. The probe pulse was a white light continuum that spanned ∼320–720 nm and was set to magic angle (54.7°) with respect to the pump to minimize artifacts from molecular rotation. The pump–probe time delay, Δ*t*, was generated by altering the optical path length of the probe, using a gold retroreflector mounted on a motorized delay stage; this enabled for a maximum Δ*t* of 2.9 ns. The pump and probe beams intersect the samples that are continuously recirculated from a 25 mL reservoir between two 25 mm CaF_2_ windows (thickness: 1 mm front, 2 mm back) in a flow cell (Demountable Liquid Cell, Harrick Scientific Products Inc., Pleasantville, NY, USA) using a diaphragm pump (SIMDOS 02). A polytetrafluoroethylene spacer, which defined the optical path length of the sample, was used between the windows. The following spacer sizes were used to ensure the optical density of the sample with the pump was <0.5:250 μm for **PDNO** in *n*-heptane and **NDNO** in DMF; 390 μm for **PDNO** in CHCl_3_, DMF, and MeOH, and **NDNO** in CHCl_3_ and *n*-heptane; and 950 μm for **NDNO** in MeOH.

To obtain dynamical information from the fs-TA results, the spectra were fitted with a global sequential $\left(A\overset{\tau_{1}}{\to }B\overset{\tau_{2}}{\to }C\overset{\tau_{3}}{\to }D\right)$ fitting model in the software package Glotaran.^27^ The model incorporates multiple sequential exponential decays convoluted with a Gaussian to model the IRF. The lifetimes produced from the fit correlate to evolution-associated difference spectra (EADS) that represent the evolving spectral features that occur on that lifetime. The residuals from the fit are used to assess the quality of the fit. The IRF for the experiments was estimated from a solvent-only scan of MeOH at a pump wavelength of 505 nm and with an optical path length of 950 μm and was found to be ∼115 fs. The TEAS spectra were chirp corrected in the software package KOALA, utilizing a third-order polynomial to model the dispersion.^28^

#### Synthesis of PDNO and NDNO

The synthesis of **PDNO** and **NDNO** was achieved as shown in Scheme S1 of the ESI. In short, to a suspension of 1.44 g (10 mmol) of β-naphthol in 10 mL of distilled water in a 50 mL round-bottom flask was added 1 g of NaOH, and the resulting solution was stirred under ice cooling. Meanwhile, a chilled suspension of 10 mmol of 1-naphthylamine/aniline suspended in 3 mL of concentrated HCl and 10 mL of water was treated with 0.5 g of NaNO_2_ dissolved in 3 mL of chilled water dropwise. The resulting solution was filtered through a glass funnel fitted with a cotton plug. The clear filtrate obtained was added dropwise to the already prepared clear alkaline solution of β-naphthol, and the solution was maintained at 0–5 °C during addition. A large amount of red/magenta solid precipitated instantly. After the addition, the solution was left to stir overnight. Then the contents of the round-bottom flask were poured into 20 mL of brine solution. The solids were filtered under suction and washed with three 10 mL portions of distilled water. The impure filter cake was suspended in 30 mL of hot absolute ethanol and stirred for 15 min. The solution was filtered under suction, and the solids were dried in air overnight. 0.959 g of dark magenta solid was obtained, which was TLC pure. Yield: 25% for **PDNO** and 32% for **NDNO**. The NMR (in CDCl_3_ solvent) and FTIR spectra are provided in Figures S1–S4.

## Results and Discussion

**PDNO** and **NDNO** were obtained in pure form by the diazo coupling reaction scheme with β-naphthol and were characterized by IR, NMR, and X-ray diffraction (single and powder) techniques. The various tautomeric forms of **PDNO** and **NDNO** are presented in Scheme 1 for ease of discussion in subsequent sections.

### Single Crystal Structure of NDNO

**NDNO** was dissolved in a binary mixture of CHCl_3_ and MeOH, and single crystals suitable for X-ray diffraction were obtained after a few days. **NDNO** crystallizes in the *P_n_* space group and *P*_–2yac_ hall group (CCDC No: 2471384). The salient features of the crystal structure of **NDNO** are presented in Figure 1. As evident, an elongated hydrogen bond exists between the atoms H_1_ and N_1_ (*d*_N2–O1_ = 2.522 Å, *d*_N2–H1_ = 1.785 Å, *d*_O1–H1_ = 0.822 Å). The unit cell of **NDNO** comprises two molecules of **NDNO**. CH···π-type nonclassical interactions lead to the formation of a cusp-shaped architecture. The crystal structure of **NDNO** is remarkably different than that of **PDNO,** as the latter is reported to exist in the hydrazo form in the single crystal form.^29^ The intramolecular hydrogen bond in **NDNO**, as seen in the crystal structure, is expected to remain intact in solvents of lower polarity due to negligible solute–solvent interaction, whereas higher solute–solvent interactions in solvents of higher polarity/proticity are expected to rupture the hydrogen bonding, which could lead to differences in photobehavior of **NDNO**.

**Figure 1:**
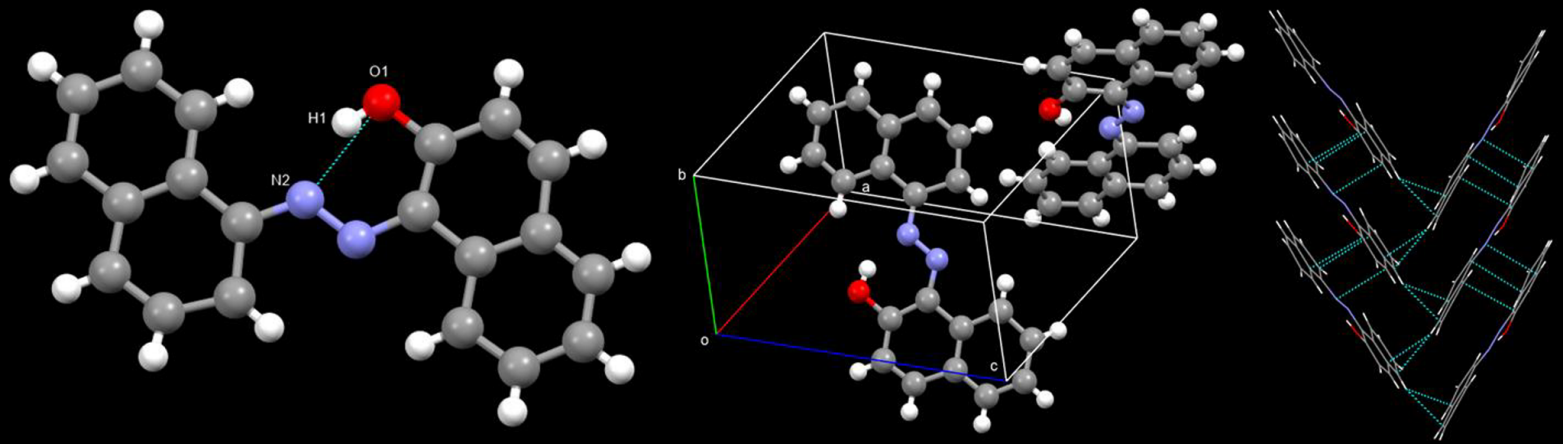
Salient features of the crystal structure of **NDNO**. (Left) Hydrogen bond present in **NDNO**, (middle) unit cell packing of **NDNO**, and (right) cusp-shaped architecture formed by supramolecular interactions.

### Steady-State and Time-Resolved Spectral Behavior in Solution

To begin with, the steady-state absorption and emission spectra of **PDNO** and **NDNO** were recorded in solvents of varying polarity and viscosity, namely, *n*-heptane (ε = 1.90, η = 0.31), CHCl_3_ (ε = 9.14, η = 0.44), DMF (ε = 20.7, η = 0.85), and MeOH (ε = 33.6, η = 0.59) (Figures 2 and 3).^30,31^ The results of the absorption spectra are discussed first. Irrespective of the solvent employed, the absorption spectrum of **NDNO** was red-shifted compared to **PDNO**. This could be a result of greater stabilization in the excited state of **NDNO** due to the presence of an additional phenyl ring in conjugation.^32–34^ The nature of the absorption spectra of **PDNO** is consistent with the literature reports, comprising broad structured bands spanning from 440 to 540 nm (see Figure 2).^19–22^

**Figure 2:**
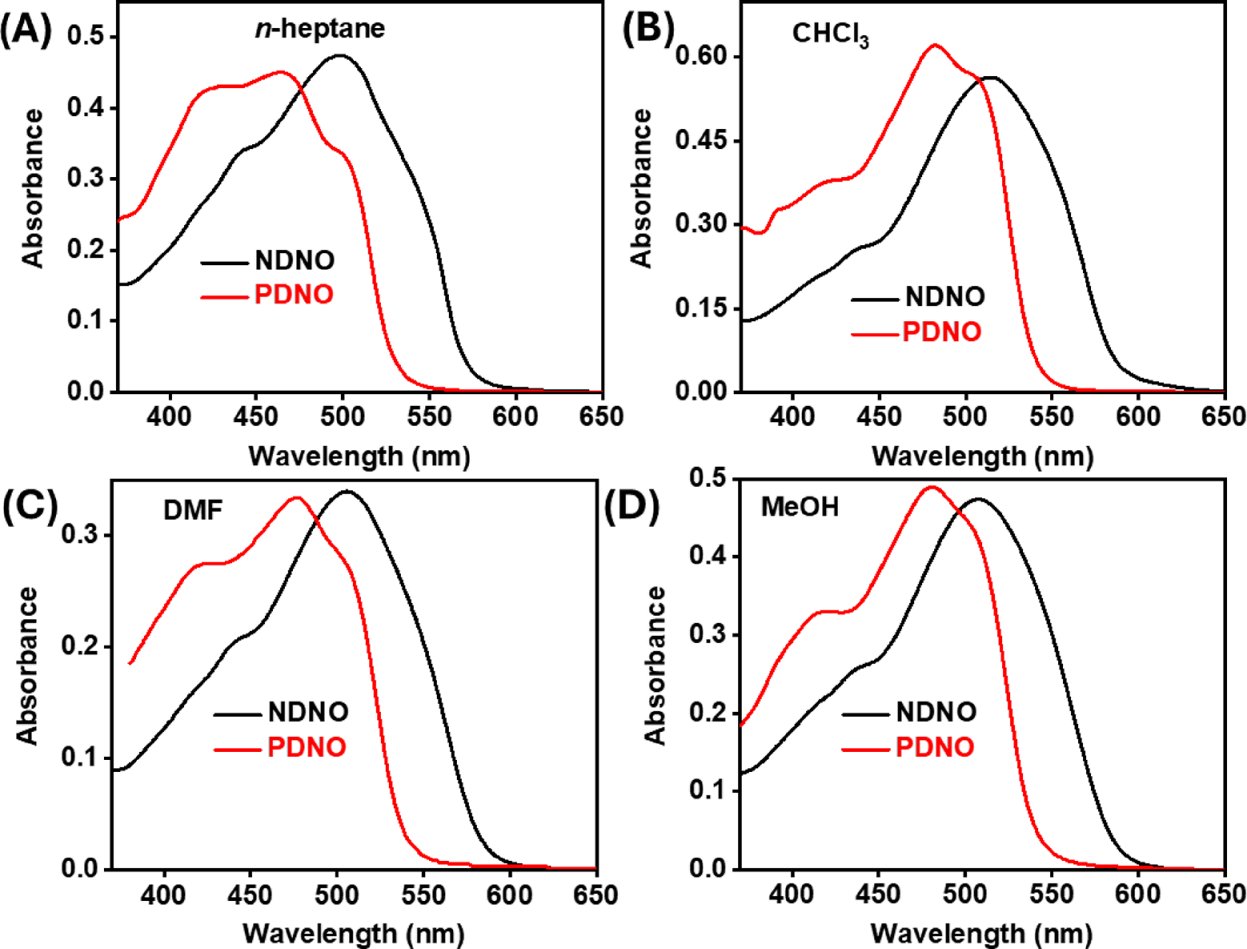
Steady-state absorption spectral profiles of **NDNO** (black) and **PDNO** (red) in (A) *n*-heptane, (B) CHCl_3_, (C) DMF, and (D) MeOH. The absorbances were made equal for the sake of comparison.

**Figure 3:**
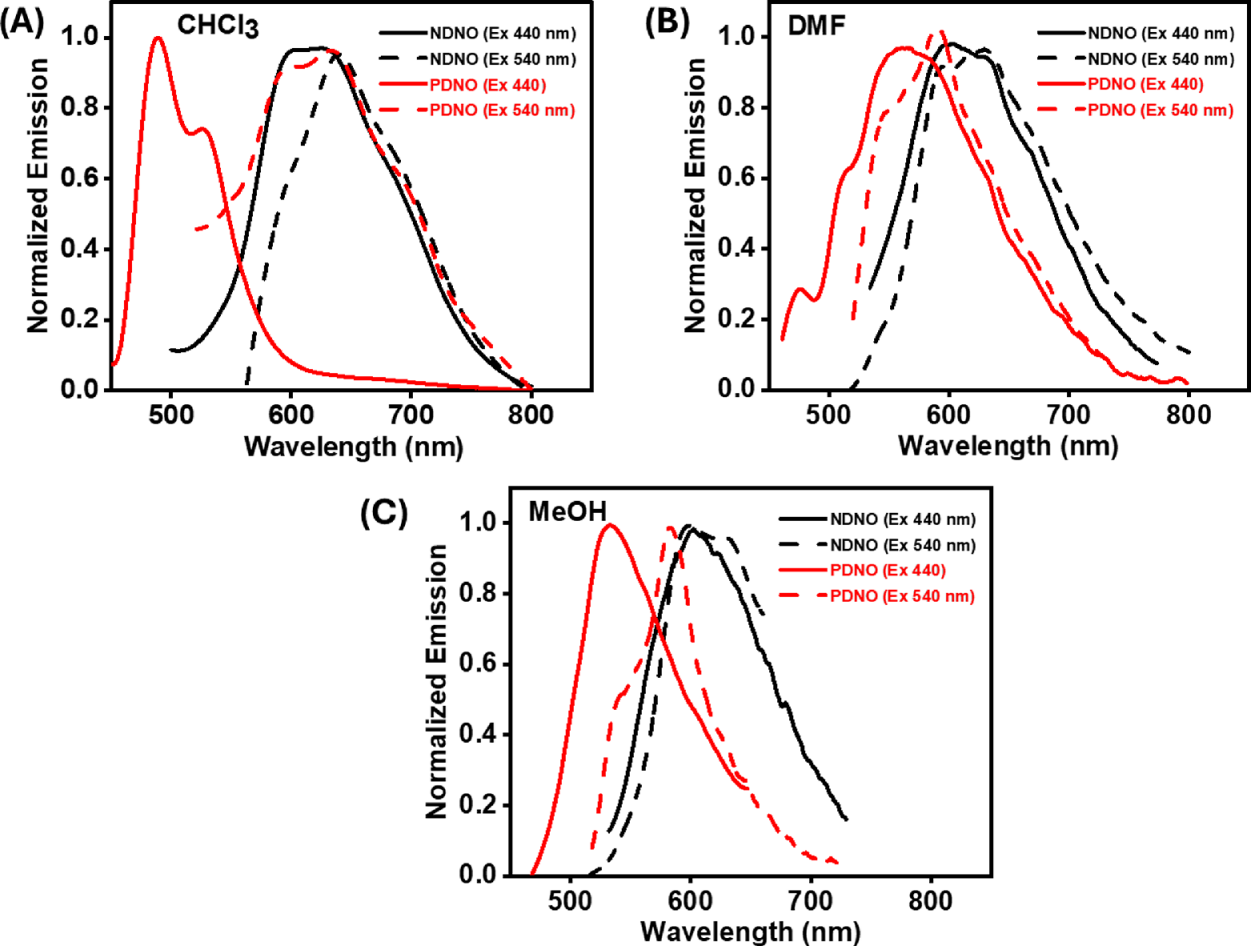
Steady-state emission spectra of **NDNO** (black) and **PDNO** (red) in (A) CHCl_3_, (B) DMF, and (C) MeOH. The excitation and emission wavelengths are mentioned in the insets. The solid lines are for **PDNO**, and the dashed lines are for **NDNO**.

The structured nature of the absorption spectra (Figure 2) is assigned to the various conformations and the corresponding π* ← π transitions of the azo and hydrazo forms of **PDNO** in solution; for the hydrazo form, the absorbance overlaps with the π* ← n transition of **PDNO**.^21^ It has been observed that the thermal isomerization from the azo to hydrazo forms of o-hydroxy azo-dyes (where the azo linkage is *ortho* to the hydroxy functionality) occurs in the ground state, albeit arrested at low temperatures.^21,29^ The presence of multiple absorbing species in the ground state is also confirmed by multipeak fitting of the experimental absorption maxima (Tables S1, S2, and Figures S4, ESI), which shows three simulated absorption bands at 415–420 nm, 470–480 nm, and 500–510 nm, which may be assigned, respectively, to π* ← π transitions in the azo and hydrazo forms as well as a π* ← *n* transition in the azo form of the two dyes.^21^ It is to be noted that the absorption spectrum of **PDNO** in MeOH lacked the broad hump (Figure 2D),^34^ which could be a possible convolution of the π* ← π and π* ← *n* transitions in MeOH, a consequence of the protic nature of the solvent.^35,36^ Nevertheless, the presence of the π* ← *n* bands in MeOH is supported by Gaussian fits to the experimental data, as can be seen in Figure S4 of the ESI.

As stated above, the absorption spectra of **NDNO** were red-shifted compared to **PDNO**. Deconvolution of the absorption peaks (Table S2, ESI Figure S5) leads to two prominent bands at 430–460 and 515–550 nm, respectively. The high-energy band is assigned to the π* ← π transition of the azo form, and the low-energy band is assigned to the hydrazo form. We may conclude that the hydrazo and the π* ← *n* transitions are energetically comparable in the case of **NDNO**, due to the absence of any third absorption peak, another consequence of the extended conjugation compared to **PDNO**.

The emission spectra of **PDNO** and **NDNO** were also recorded. As the deconvoluted absorption peaks showed the existence of multiple absorbing species, the dyes were excited at 440 and 540 nm, respectively (Figure 3). It is worth mentioning that the emission spectra were too weak in terms of intensity to be recorded in *n*-heptane at room temperature for any of the dyes. We discuss our findings in different solvents below.

In CHCl_3_, upon excitation of **PDNO** at 440 nm, dual emission bands were observed at 500 nm (Stokes’ Shift 2727 cm^–1^) and 650 nm (Stokes’ Shift 7343 cm^–1^) (Figure 3A, solid lines). The higher value of Stokes’ Shift for the 650 nm emission band supports the excited state tautomerization of the *trans*-azo form to the *trans*-hydrazo form, in keeping with literature reports.^19,20^ The value of the Stokes’ shift of the 500 nm emission band was consistent with literature reports of emission from the locally excited *trans*-azo form of **PDNO**.^22^ Upon excitation of **PDNO** at 540 nm, only the lower energy band at ∼650 nm was observed with a Stokes’ Shift value of 3133 cm^–1^ that is consistent with emission from the excited *trans*-hydrazo forms of **PDNO** (Figure 3A, red dashed line). For **NDNO**, no dual emission was observed upon excitation at 440 or 540 nm (Figure 3A). The relatively low energy displacement of the emission from both the *trans*-azo and *trans*-hydrazo forms of **NDNO** compared to **PDNO** could be rationalized by considering the effect of increased conjugation, as already reflected in the absorbance spectra (Figure 2).

In DMF, (Figure 3B), the convolution of the emission bands in **PDNO** could be an outcome of the lowering of the energy gap between the *trans*-azo and *trans*-hydrazo forms in DMF. However, the change in solvent from CHCl_3_ to DMF produces little effect on the emission spectrum of **NDNO**.

In MeOH, the emission spectrum of **PDNO** shows distinct emission wavelengths upon change in the excitation wavelength, similar to that seen in CHCl_3_ (Figure 3C). In line with emission in CHCl_3_ and DMF, the emission maxima of **NDNO** showed little difference upon a change in the excitation wavelength.

The analyses of the fluorescence excitation spectra are revealing, providing insight into the nonemissive nature of the π* ← *n* transition (ESI, Figure S6). For **PDNO**, the fluorescence excitation spectra when recorded against observation wavelengths of 550 nm and 600 nm in different solvents (Figure S6A–C). In chloroform, the excitation spectra were observed to be blue-shifted compared to the corresponding absorption spectrum. However, in MeOH, a perfect superposition of the absorbance and excitation spectra was observed (Figure S6). This could be rationalized by considering the presence of the π* ← *n* band along with the azo and hydrazo forms of **PDNO**, the former being essentially nonemissive, as supported by the literature.^21^ Hence, the emission spectra observed are caused by the deexcitation of the azo and hydrazo forms to the ground state. The π* ← *n* transition is arrested in the protic solvent MeOH as already described earlier, leading to a better superposition. For **NDNO**, however, the difference between the fluorescence excitation and absorption maxima was negligible (Figure 4D,E). This observation supports the assumption of the π* ← *n* transition being energetically closer to the hydrazo form in **NDNO** compared to that in **PDNO** (vide supra). It is to be noted that the fluorescence excitation spectra could not be collected at the same wavelength values in all solvents, owing to the poor emission signal.

**Figure 4:**
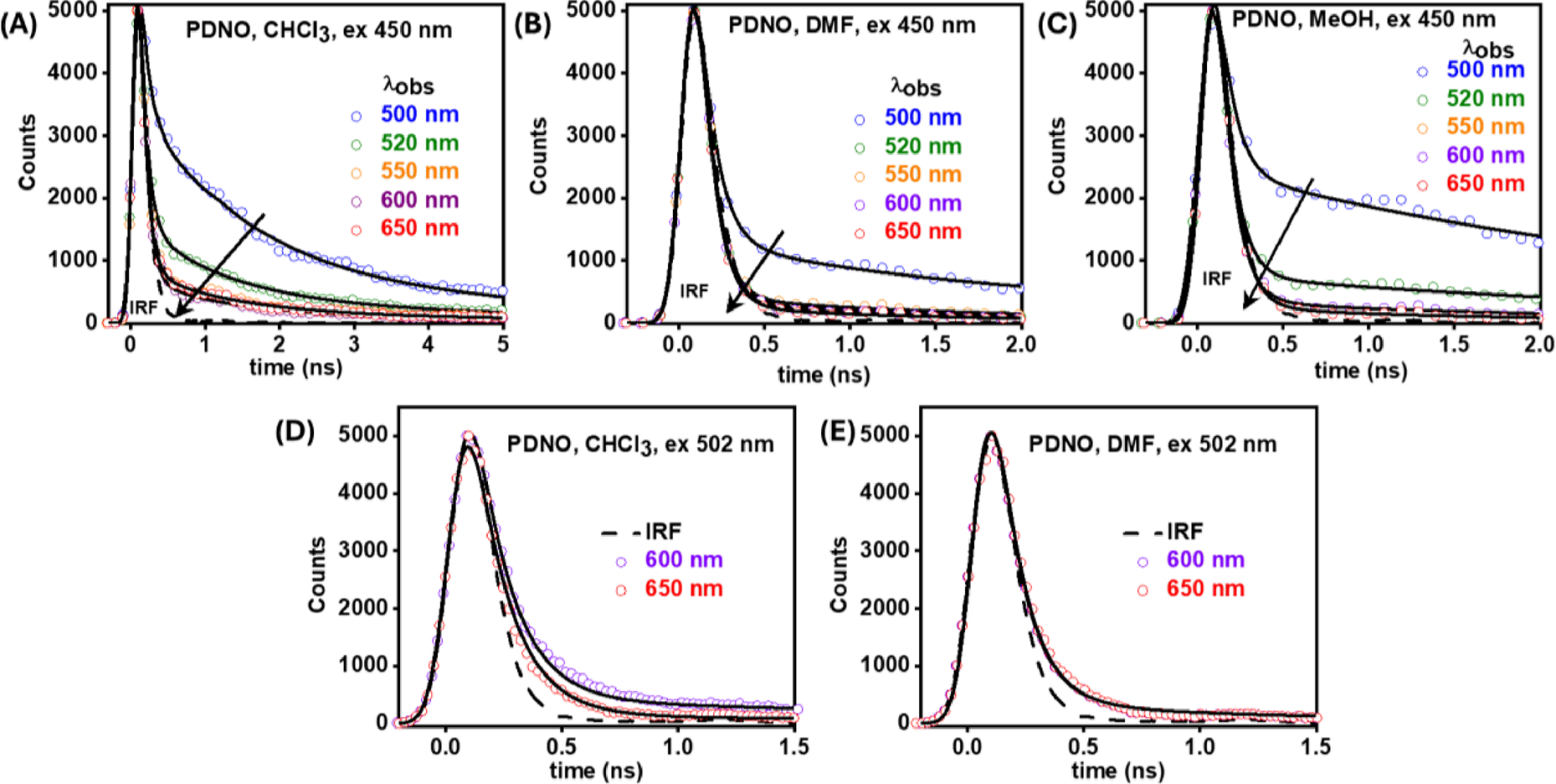
Magic angle emission lifetime decays of **PDNO** when excited at (A–C) 450 nm and (D,E) 502 nm. The dashed line represents the IRF, and the black lines denote the best multiexponential fit to the lifetime decays. The values of the observation wavelengths are provided in the insets of each panel.

### ps-ns Emission Lifetime Decay Behavior in Solution

The absorption and emission studies of **PDNO** and **NDNO** in solution revealed the existence of the azo and hydrazo tautomers of the dyes and their differential optical behavior, in keeping with previous literature. However, the dynamic nature of the process of tautomerism from azo to hydrazo was yet to be probed. With this in mind, emission lifetime decay traces of the dyes were recorded in CHCl_3_, DMF, and MeOH by exciting at both 450 and 502 nm and observing at various emission wavelengths (Figures 4, 5, and Tables S3–S6, ESI). Photoexcitation at 450 nm excites *both* the azo and hydrazo tautomers in both **PDNO** and **NDNO**; photoexcitation at 502 nm preferentially excites *only* the hydrazo tautomers. The results of **PDNO** shall be discussed first, followed by **NDNO**.

**Figure 5:**
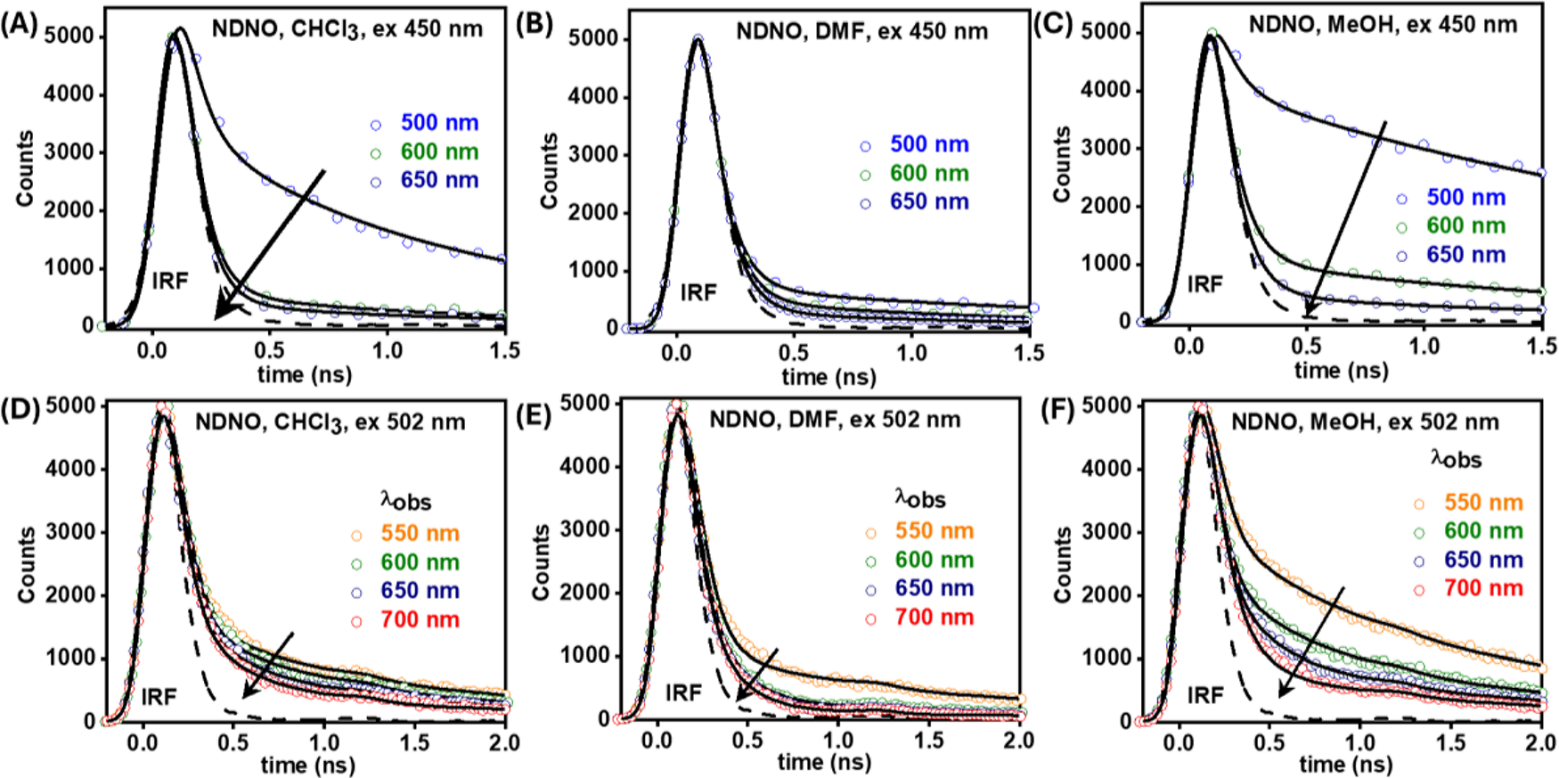
Magic angle emission lifetime decays of **NDNO** when excited at (A–C) 450 nm and (D–F) 502 nm. The dashed line represents the IRF, and the black lines denote the best multiexponential fit to the lifetime decays. The values of the observation wavelength are provided in the insets of each panel.

#### Photoexcitation of **PDNO** (*Both* Azo and Hydrazo Tautomers) at 450 nm

The decay in emission intensity, across all solvents, was fit with a global model utilizing three exponential decay functions (Figure 4A–C, Table S3, ESI). At various observation wavelengths (λ_obs_) from 500 to 650 nm, the fit furnished three lifetime components: τ_1_ = 75–90 ps, τ_2_ = 1.2–2.0 ns, and τ_3_ = 4.0–4.5 ns. The τ_1_ component has the largest contribution to the excited state population decay, and the relative amplitude of this lifetime component increases in the red λ_obs_. The τ_2_ and τ_3_ components show a maximum contribution at the blue region and a negligible contribution at the red region of observation; these components also showed a near-zero contribution in solvents of higher polarity/viscosity (DMF and MeOH) (Table S3, ESI).

#### Photoexcitation of **PDNO** (*Only* the Hydrazo Tautomer) at 502 nm

The decay in emission intensity, across all solvents, was fit with a global model utilizing two exponential decay functions (Figure 4D,E, Table S4, ESI). The extracted lifetime components were τ_1_ = 100–120 ps and τ_2_ = 1.2–1.3 ns. τ_1_, similarly to photoexcitation at 450 nm, showed the greatest relative contribution (Table S4, ESI). To note, for MeOH, decays could not be recorded due to very low signals obtained upon excitation at 502 nm.

#### Photoexcitation of **NDNO** (*Both* Azo and Hydrazo Tautomers) at 450 nm

The decay in emission intensity, across all solvents, was fit with a global model utilizing three exponential decay functions (Figure 5A–C, Table S5, ESI), similar to **PDNO**. The extracted lifetime components were τ_1_ = ∼80 ps, τ_2_ ∼ 1.0 ns, and τ_3_ = 3.5–6.0 ns. The trend in the amplitude of each lifetime component in each solvent was similar to that of **PDNO**.

#### Photoexcitation of **NDNO** (*Only* the Hydrazo Tautomer) at 502 nm

The decay in emission intensity, across all solvents, was fit with a global model utilizing two exponential decay functions (Figure 5D–F, Table S6, ESI), yielding the following lifetimes: τ_1_ = 130–150 ps and τ_2_ = 1.5–2.0 ns. The variational trend of the amplitude of the lifetime components was, once again, similar to that described for **PDNO** (Table S6, ESI).

As the trends in the emission lifetimes were similar for both dyes, suggesting that their dynamics are driven by the same mechanism, a probable spectrodynamic model can be proposed to explain the picosecond-nanosecond emission spectrodynamic behavior, invoking ESIPT of the hydroxy azo dyes. At the outset, it is worthwhile to consider the thermal interconversion of the azo to hydrazo forms of the dyes in solution, resulting in the simultaneous presence of both tautomers in the ground state as reported previously.^19,37^ The azo form absorbs at shorter wavelengths than the hydrazo form, irrespective of whether the tautomer is in its *cis* or *trans* conformer. Hence, irradiation at 450 nm photoexcites both the azo and hydrazo forms due to the overlapping nature of their absorption (Figure 1), and the hydrazo forms are preferentially excited upon irradiation at 502 nm. Importantly, although each of the azo and hydrazo tautomers could exist freely in their *cis* and *trans* forms, as depicted in Scheme 1, the *trans* form of each tautomer is more stable owing to greater stabilization from the extended conjugation in the *trans* conformer.

When excited at 450 nm, the *cis* and *trans* isomers of the azo and hydrazo forms of both dyes are excited (see above, noting that the *trans* form dominates). As the conversion of the azo to hydrazo form in the excited state is reported to be ultrafast,^19^ it is reasonable to assume that the azo tautomer converts to its hydrazo form, followed by a fast deactivation in the ps-ns regime. The emission intensity of the hydrazo form is maximum at the longest λ_obs_ of the steady-state emission spectrum (Figure 2). Hence, the increasing contribution of the τ_1_ component at the red λ_obs_ leads us to assign it as the lifetime of deactivation of the *trans*-hydrazo-tautomer, as the ultrafast photo tautomerism yields the *trans*-hydrazo from the excited *trans*-azo form. Concurrently, the twisting around the hydrazo linkage (Scheme 1) in the excited state is likely leading to the formation of the excited *cis*-hydrazo form of the tautomer, given the ultrafast twisting motion associated with the *cis*-*trans* isomerization.^20,21^ Emission from the *cis*-hydrazo tautomer is reported to be slower compared to the *trans-*conformer.^19^ Hence, the component τ_2_ was assigned to the lifetime of the *cis*-hydrazo tautomers. Finally, the slowest component (τ_3_) that contributes least to the excited state population could be attributed to the de-excitation of the excited *trans*-azo tautomers. However, it is plausible that the observed lifetime could be due to deactivation of both the *trans* and *cis* azo tautomers, given similar radiative decay times.

On the other hand, when excited at 502 nm, the deactivation is anticipated to occur solely from the excited hydrazo-tautomers (see above), hence two lifetime components are obtained.

The overlapping emission bands of the various azo and hydrazo conformers make it difficult to assign separately the *cis* and *trans* conformers in the steady-state profile (Figure 3). However, the picosecond-to-nanosecond lifetime decay analyses point toward the presence of the *cis-* and *trans*-conformers of the tautomeric forms of both dyes in solution. In addition, from the greater contributions of the τ_1_ component, it could be realized that the radiative deexcitation of the *trans*-hydrazo tautomer was favored, irrespective of the solvent nature. A proposed photophysical scheme illustrating the tautomeric processes occurring in **PDNO** and **NDNO** after photoexcitation is shown in Scheme 2.

**Scheme 2:**
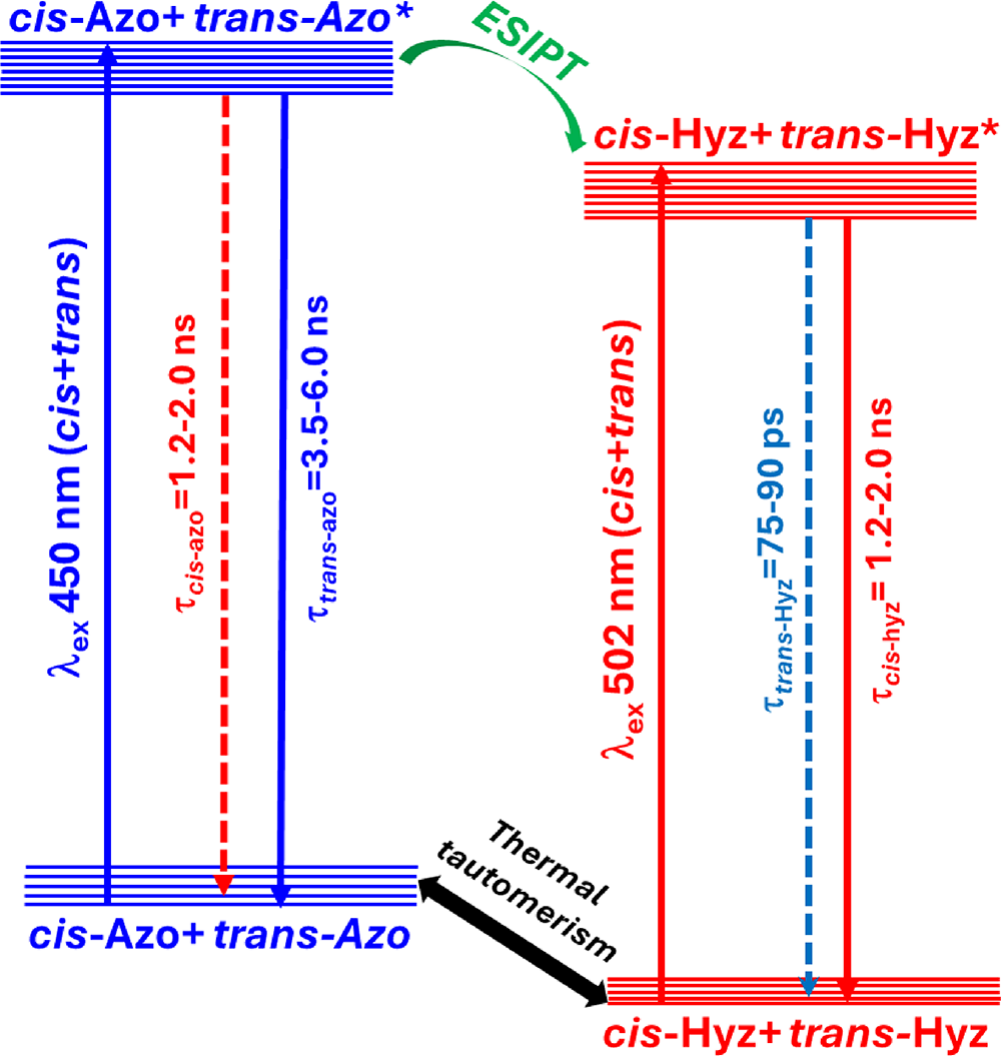
Collective Photophysical Scheme of **PDNO** and **NDNO** after Photo-Excitation (Not to Scale)^a^

a For visualization of the conformers of the tautomers, refer to Scheme 1.

At this stage, we deemed it necessary to provide a brief discussion on the assignment of the lifetime values of the tautomeric forms. Although the excited-state tautomerism from the azo to hydrazo form is ultrafast, it is not the exclusive process occurring in the excited state. Apart from that, literature reports suggest that usually the emission lifetime of the “enol” or “normal” form of a fluorophore is considerably greater than the “keto” or “proton-transferred” form, the trend encompassing a large number of PT active probes.^18,19,25,38,39^ Hence, although the photoconversion is ultrafast, it does not necessarily warrant a longer lifetime of emission from the proton transferred form.

### fs-TA Studies in Solution

The fluorescence emission lifetime values of the hydrazo tautomers of both dyes returned τ_1_ = 75–90 ps (vide supra). To investigate these photo induced processes further, we carried out fs-TA measurements in solution.

To begin with, the fs-TA heatmaps of **PDNO** and **NDNO** are presented in Figure 6 in CHCl_3_; fs-TA measurements in *n*-heptane, DMF, and MeOH are presented in Figures S7 and S8 in the ESI. Transient slices at 1.0 ps pump–probe time delay in various solvents and the EADS in CHCl_3_ of both **PDNO** and **NDNO** are presented in Figure 7; EADS in *n*-heptane, DMF, and MeOH are presented in Figures S9 and S10 of ESI. Irrespective of the nature of the solvent, the fs-TA spectra consist of four spectral features (Figures 6, S7, and S8, ESI). For ease of discussion, they shall be denoted by features I, II, III, and IV, respectively. Features I and III are positive and appear at ∼350–450 nm and ∼525–600 nm, respectively, for **PDNO**, and ∼350–450 nm and ∼550–675 nm, respectively, for **NDNO**. Features II and IV are negative, with II appearing at ∼500 nm for both molecules and IV appearing at >600 nm for **PDNO** and ∼700 nm for **NDNO**. Since Feature II for both dyes resembles the negative steady-state absorption spectrum of **PDNO** and **NDNO**, it is assigned as the ground state bleach (GSB). Features I and III are assigned to excited state absorption (ESA) to higher lying electronic excited states. Feature IV appears in a spectral region similar to the emission spectra in Figure 3 and is assigned to stimulated emission (SE).

**Figure 6:**
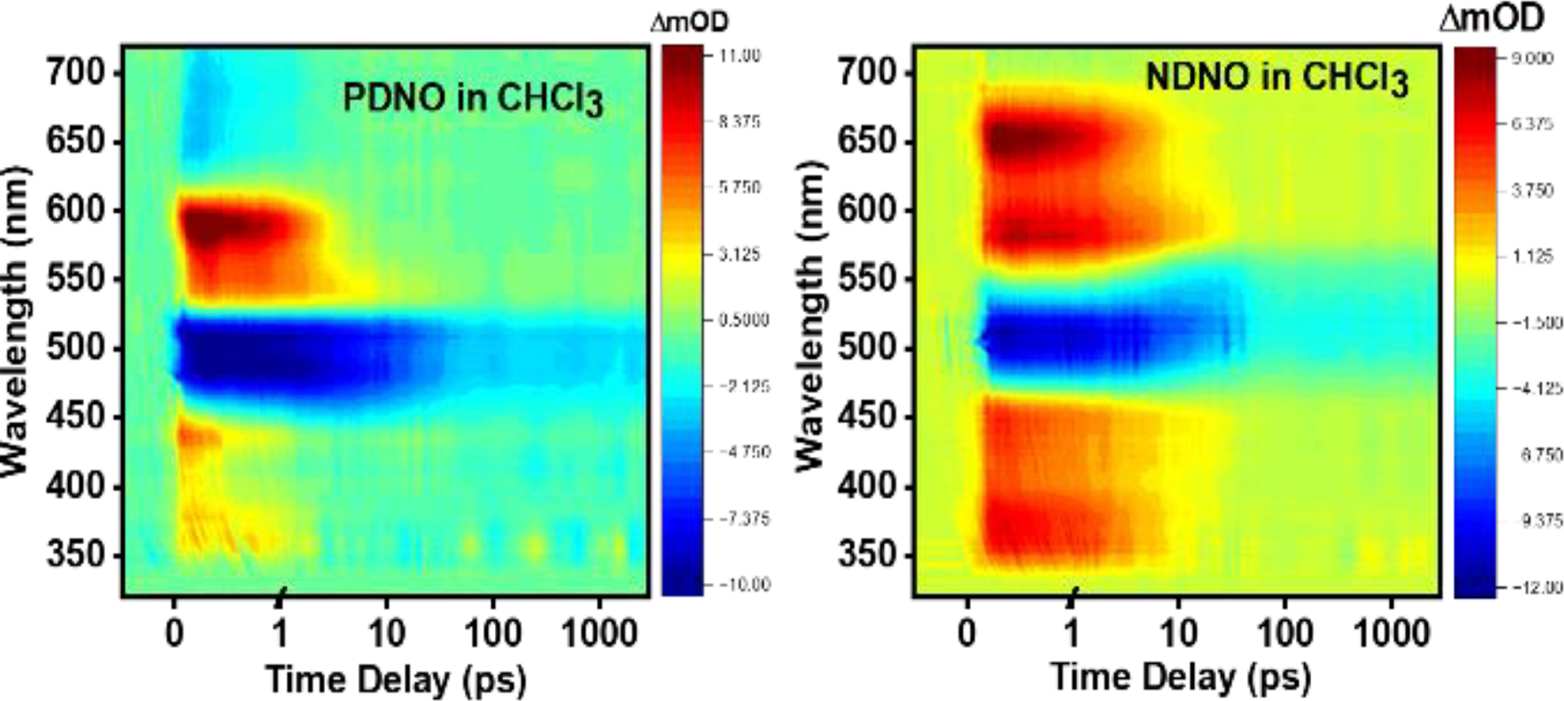
fs-TA heatmaps of **PDNO** and **NDNO** in CHCl_3_ following photoexcitation at 480 and 505 nm, respectively. The time delay is linear up to 1 ps and logarithmic from 1 to 2900 ps.

**Figure 7:**
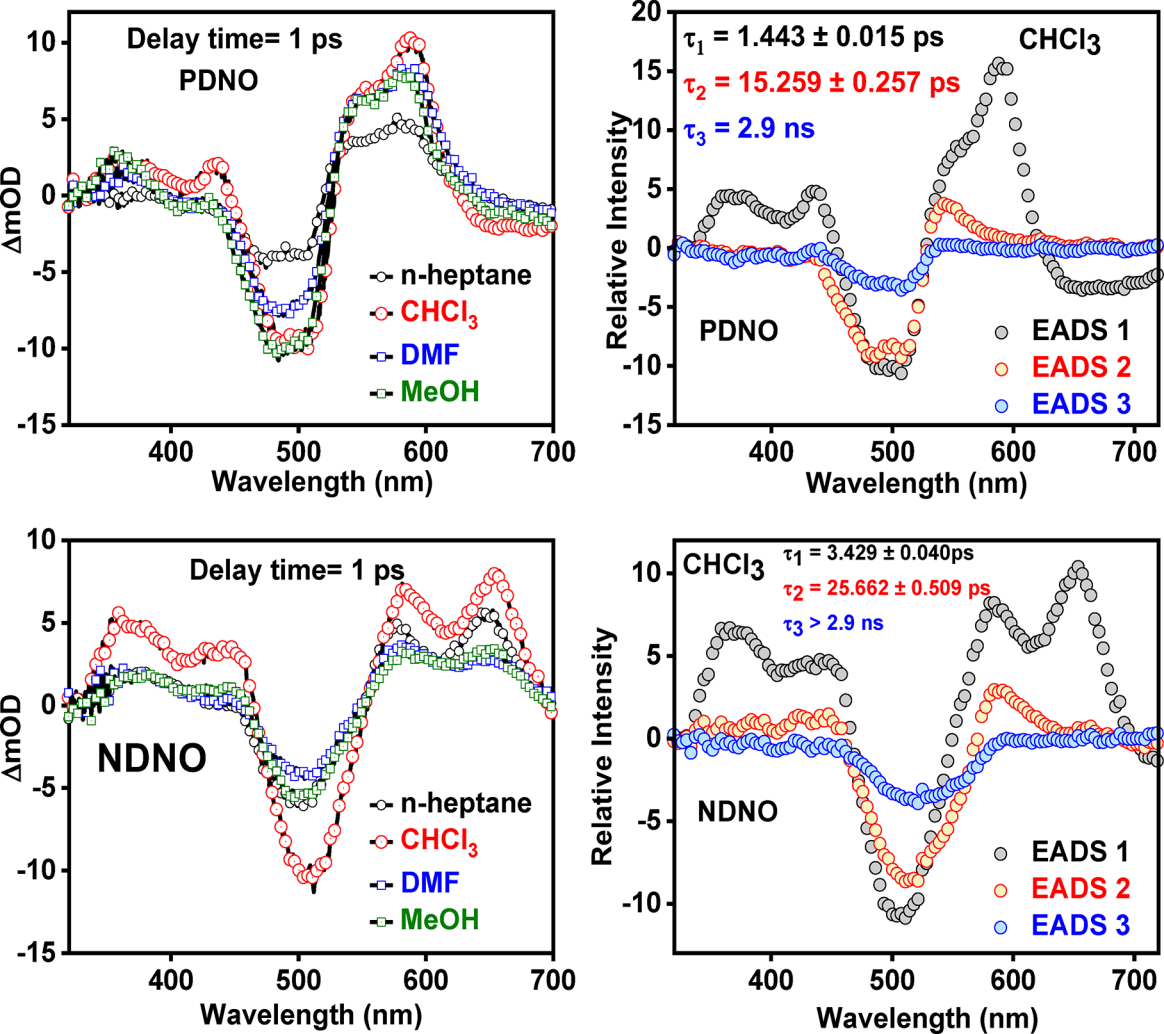
fs-TA spectra of **PDNO** and **NDNO** in various solvents at 1.0 ps delay time following photoexcitation at 480 and 505 nm, respectively, presented top left for **PDNO** and bottom left for **NDNO**. The EADS in CHCl_3_ obtained from the global sequential fitting model in the Glotaran software package (see the Experimental Section for further details) are shown in the top right for **PDNO** and the bottom right for **NDNO**. The corresponding lifetimes of the sequential fits for PDNO and NDNO in CHCl_3_ are provided in the top right and bottom right panels (quoted errors relate to two standard deviations of the fitting).

Due to the strong spectral overlap of the tautomeric forms in the ground state irrespective of the solvents, as well as simultaneous excitation of both species under the experimental conditions performed, an exponential fitting of the signals would render the analysis of each signal region intractable. Hence, the spectra were fitted with a global sequential fitting model returning EADS that represents the evolving spectra that occur on the corresponding lifetimes (Figures 7, S7, and S8 in ESI). Akin to the fs-TA heatmaps, the EADS of both **PDNO** and **NDNO** shows a similar qualitative pattern. The presence of multiple species prevents detailed assignment of each time component and should be considered as a representative time scale associated with one (or multiple) photochemical and/or photophysical processes.

The first extracted lifetime τ_1_ (with associated EADS 1) is the same order of magnitude (1.0–2.0 ps) for both dyes, irrespective of the solvent. This time scale is assigned to the vibrational cooling (VC) of the excited azo and hydrazo tautomers; we draw confidence in this assignment due to the similarity of τ_1_ to commonly reported values of VC of organic chromophores.^40^ The second extracted lifetime τ_2_ (with associated EADS 2), ranging between 5.0 and 12.0 ps, is plausibly assigned to *trans*-*cis* isomerization of the dyes, in line with previously reported studies on **PDNO**.^22^ Lastly, EADS 3, corresponding to τ_3_, a lifetime longer than the maximum time window of the experiment (2.9 ns), manifests mainly as a red-shifted GSB feature. This indicates possible long-lived ground state depletion of the hydrazo tautomer through either trapped excited state population of the hydrazo tautomer or preferential reformation of the azo tautomer’s electronic ground state. A long-lived excited state population is not unexpected due to the presence of a π* ← *n* transition in the dyes, which are reported to have long nonradiative deexcitation time scales.^41–45^

It is to be noted that the values of the time constants show an interesting trend in terms of both the solvent chosen and the annulation of the dyes. First, we observed that the lifetimes of the relaxation processes are considerably slower in chloroform compared to other solvents (Figure 7, ESI, Figures S9 and S10) for both **PDNO** and **NDNO**. This could be rationalized by considering the combined effect of the viscosity and polarity. The reason could be attributed to the balance between polarity and viscosity and lack of protic interactions in chloroform, as shall be discussed now. The high polarity of MeOH is associated with protic interactions with the dye molecules, which leads to faster nonradative relaxation, reflected in the lifetime values (Figure 7, ESI, Figures S9 and S10).^46^ On the other hand, the higher viscosity in DMF is associated with a lower value of the dielectric constant, leading to the lower value of the relaxation lifetime.^47^ Thus, we could safely state that chloroform provides the optimal polarity and viscosity to reduce the rate of relaxation of the dyes.^48^ Second, the relaxation time for **NDNO** was longer than **PDNO** in all solvents. We attribute this difference based on the greater conjugation in **NDNO** due to the presence of an extra phenyl ring, which is reported to have slower relaxation pathways in organic dyes.^49,50^ Hence, the fs-TA studies provided insights into the dependence of nonradiative relaxation processes of the azo and hydrazo tautomers of the dyes on the nature of the solvent and the annulation of the dye molecule.

## Conclusions

The present study explored the excited state dynamics of two azo dyes, **PDNO** and **NDNO**, in solvents of varying polarities as well as viscosities using a combination of both steady-state and time-resolved experiments. From the steady-state and picosecond-nanosecond emission decay analyses, we infer that the ESIPT process in both **PDNO** and **NDNO** is considerably fast, occurring at a time scale less than 100 ps. The azo and hydrazo tautomeric forms of **PDNO** and **NDNO** are present in the ground state; the thermally allowed conversion of the azo to the hydrazo form results in a very poor quantum yield of the dyes in solution phase. From the fs-TA studies, it was observed that the transient relaxation pathways are considerably slowed in the presence of chloroform as well as through the addition of an extra phenyl ring (in **NDNO**). The current study provides an in-depth perspective of the combined emissive and nonemissive decay pathways associated with azo dyes as well as the effects of viscosity and annulation.

## Supplementary Material



## Notes

Synthetic scheme, characterization, tables for various steady state and time-resolved experiments, excitation profiles, and detailed EADS of PDNO and NDNO in various solvents (PDF)
