# Supplementary material for: Interrogating the Azo-Hydrazo Proton Transfer Process in the Excited State Using a Multipronged Spectroscopic Study: Effect of Annulation and Solvent
Source: ACS Phys Chem Au. 2025 Sep 11;5(6):649–59. doi: 10.1021/acsphyschemau.5c00067 (PMC12670284; doi:10.1021/acsphyschemau.5c00067)
Supplement: Supplementary file 1 [file pg5c00067_si_001.pdf]

Electronic Supporting Information

Interrogating the Azo-Hydrazo Proton Transfer Process in the Excited State using a Multipronged Spectroscopic Study: Effect of Annulation and Solvent

Jack Dalton, [a] Vasilios G. Stavros, *[a] Arghyadeep Bhattacharyya*[b]

[a] School of Chemistry, University of Birmingham, Edgbaston, Birmingham, B15 2TT, UK

[b] Department of Chemistry, Tripura University (A Central University), Suryamaninagar-799022, India
*Corresponding author(s)

E-mail: arghyadeepbhattacharyya@tripurauniv.ac.in, v.stavros@bham.ac.uk

Contents

1. Synthetic Scheme of the dyes.

2. ^{1}H NMR of PDNO.

3. ^{1}H NMR of NDNO.

4. ^{13}C NMR of NDNO.

5. FTIR spectra of PDNO and NDNO.

6. Tables for Steady-State spectral parameters for PDNO & NDNO.

7. Gaussian fits provided to the experimental absorption profiles of PDNO & NDNO in various solvents.

8. Excitation profiles of PDNO and NDNO in various solvents.

9. Tables for emission lifetime values of PDNO and NDNO in various solvents.

10. Chirp corrected fs-TA heatmaps of PDNO & NDNO in various solvents.

11. EADS of PDNO and NDNO in various solvents.

1. Synthetic Scheme of the dyes.

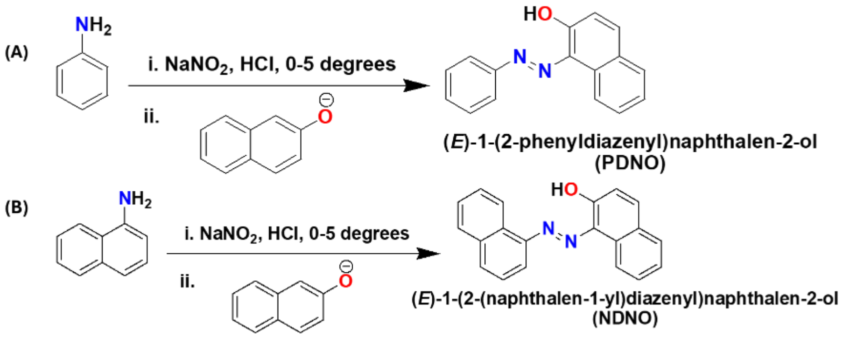

Scheme S1. Synthetic pathway of **PDNO** and **NDNO**.

2. ^{1}H NMR of PDNO.

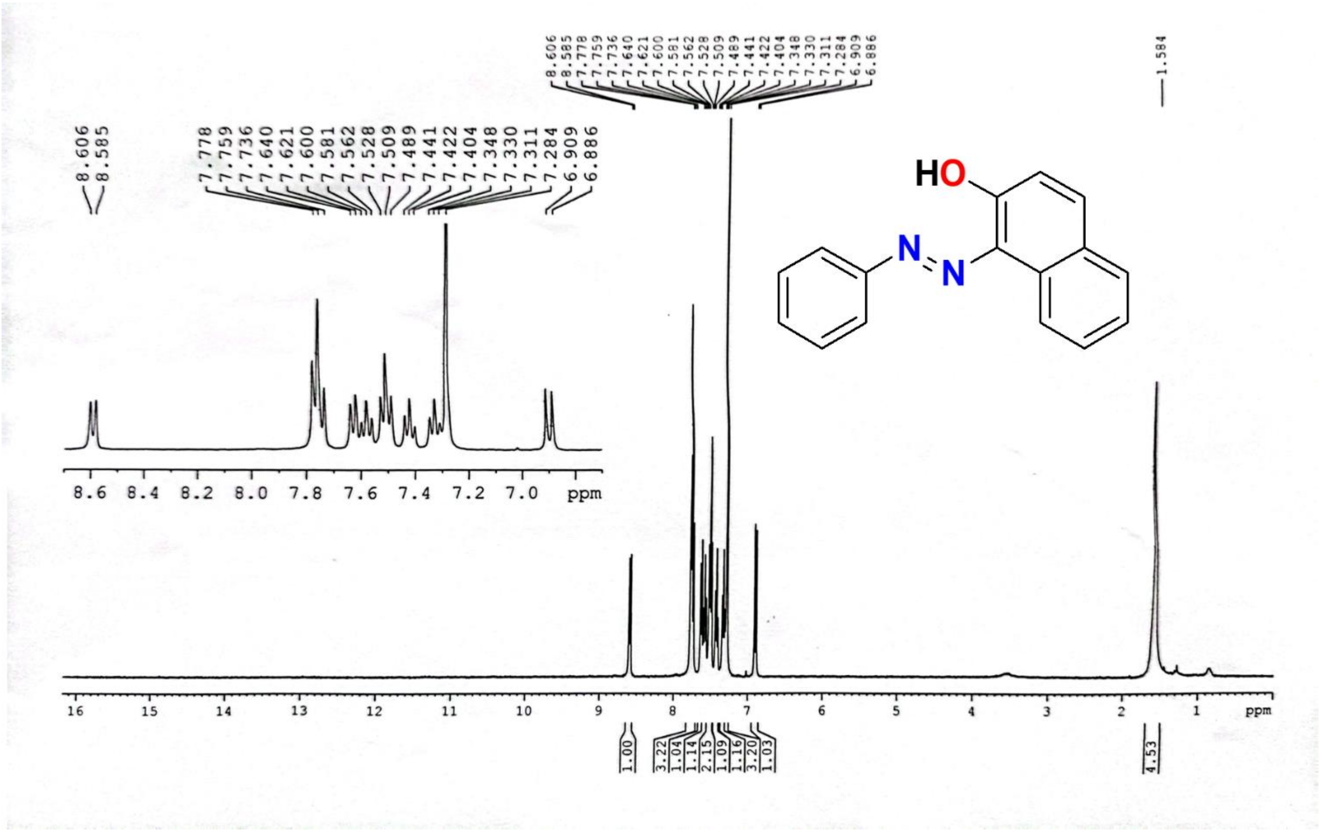

Figure S1. ^{1}H NMR of **PDNO** in $CDCl_3$.

3. ^{1}H NMR of NDNO.

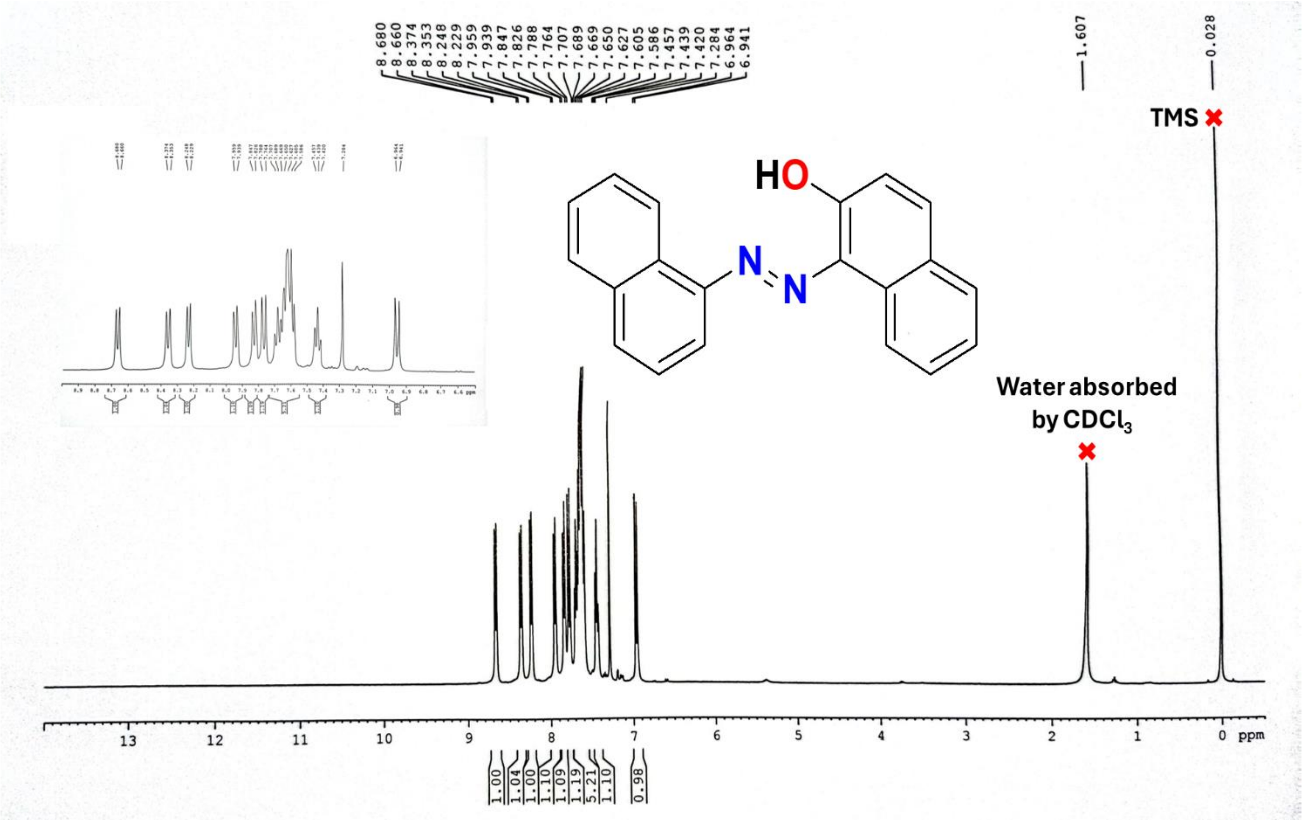

Figure S2. ^1H NMR of **NDNO** in $CDCl_3$.

4. ^{13}C NMR of NDNO.

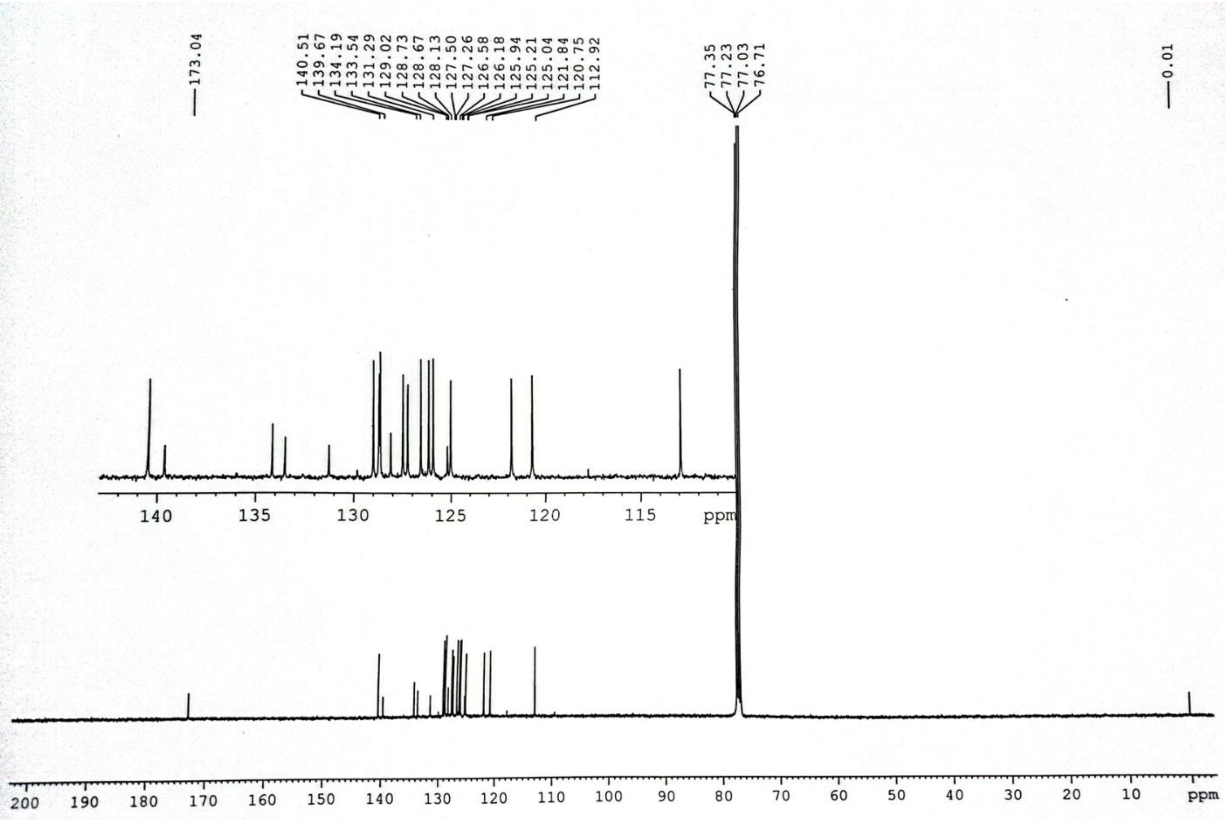

Figure S3. ^{1}H NMR of **NDNO** in CDCl$_3$.

5. FTIR spectra of PDNO and NDNO.

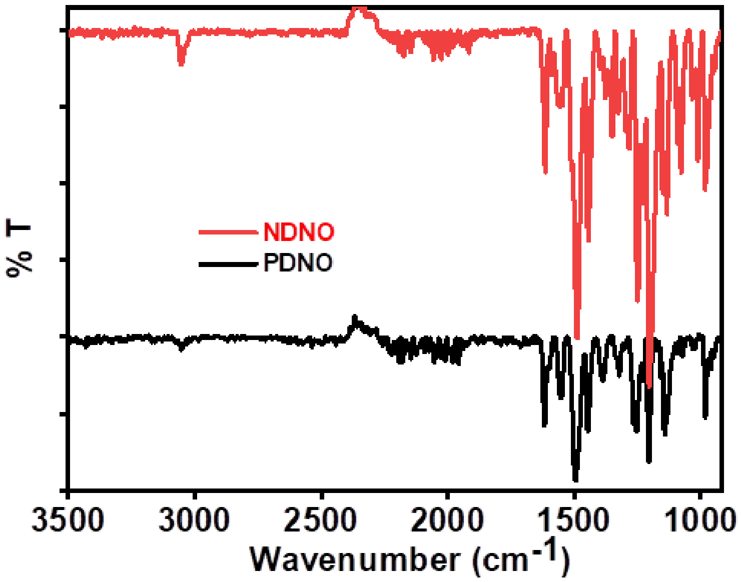

Figure S4. FTIR spectra of **PDNO** and **NDNO**.

6. Tables for Steady-State spectral parameters for PDNO & NDNO.

Table S1. Table of absorption spectral bands of **PDNO** in various solvents and the corresponding fitted values of the absorption maxima.

**Solvent**	**Expt. Abs. (nm)**	**Fitted Abs. (nm)**	$\varepsilon_1:\varepsilon_2:\varepsilon_3$
***n*-Hept**	419, 464, 502	425, 478, 507	1.2:1.4:1
**$CHCl_3$**	420, 483, 511	420, 485, 515	1:1.8:1.7
**DMF**	421, 477, 509	422, 484, 513	1:1.2:1
**MeOH**	416, 478, 507	417, 482, 512	1:1.4:1.2

Table S2. Table of absorption spectral bands of **NDNO** in various solvents and the corresponding fitted values of the absorption maxima.

**Solvent**	**Expt. Abs. (nm)**	**Fitted Abs. (nm)**	$\varepsilon_1:\varepsilon_2:\varepsilon_3$
**n-Hept**	440, 500, 548	443, 506, 546	1.1:1.6:1
**$CHCl_3$**	437, 514	460, 524	1:2.5
**DMF**	442, 504	426, 515	1:1.4
**MeOH**	438, 506	437, 515	1:1.8

7. Gaussian fits provided to the experimental absorption profiles of PDNO & NDNO in various solvents.

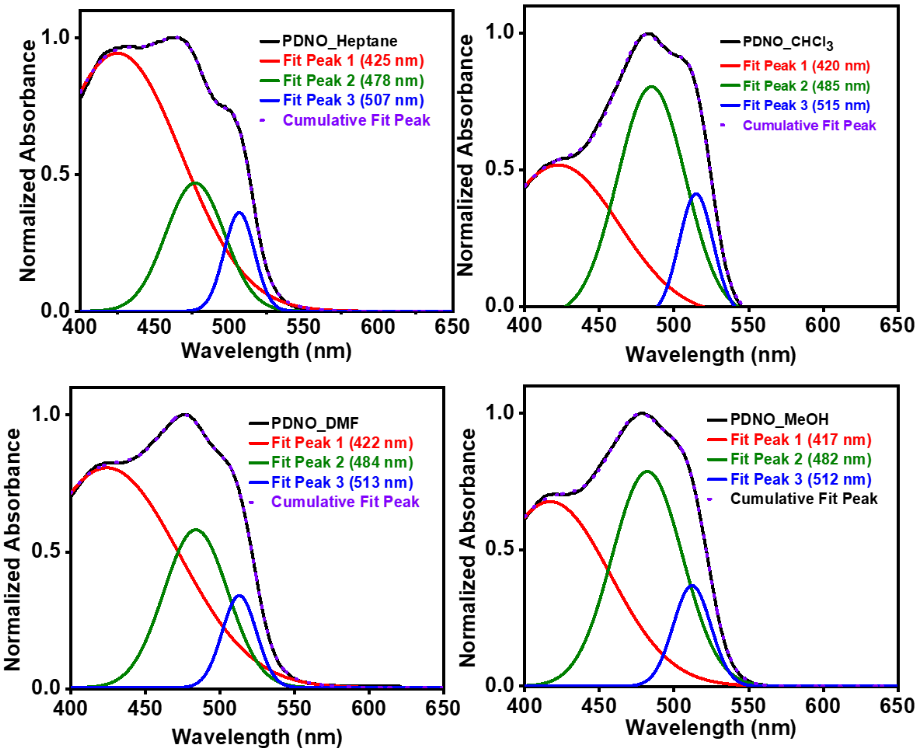

Figure S4. Gaussian fits provided to the experimental absorption profiles of **PDNO** in various solvents.

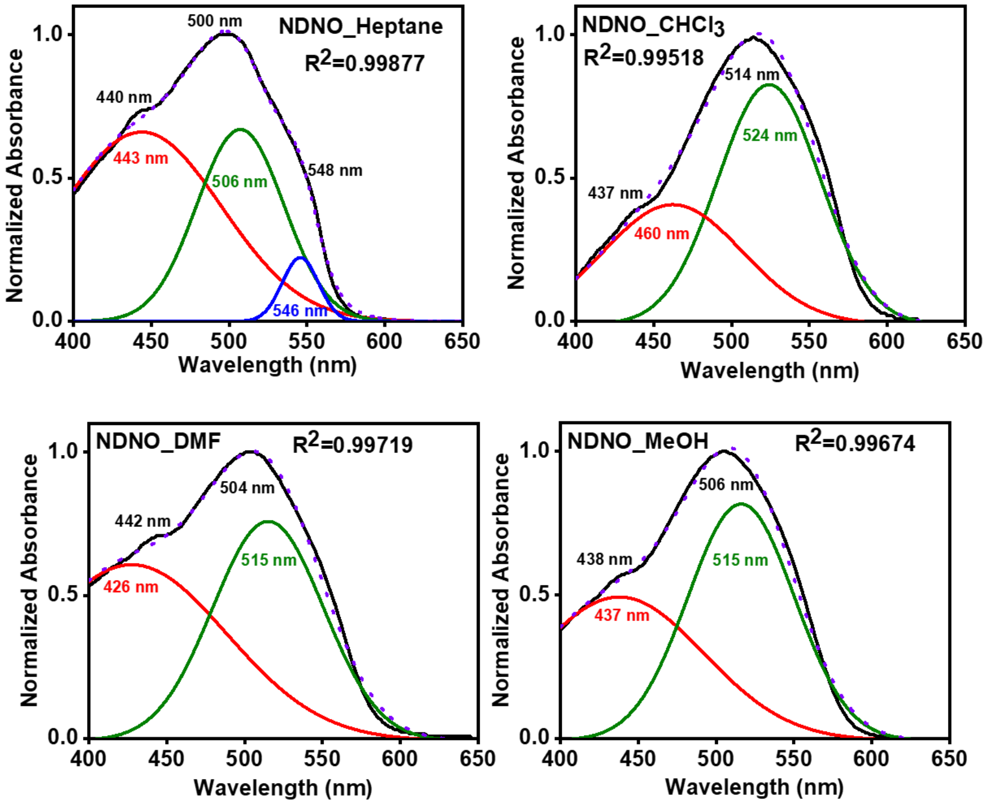

Figure S5. Gaussian fits provided to the experimental absorption profiles of **NDNO** in various solvents.

8. Excitation profiles of PDNO and NDNO in various solvents.

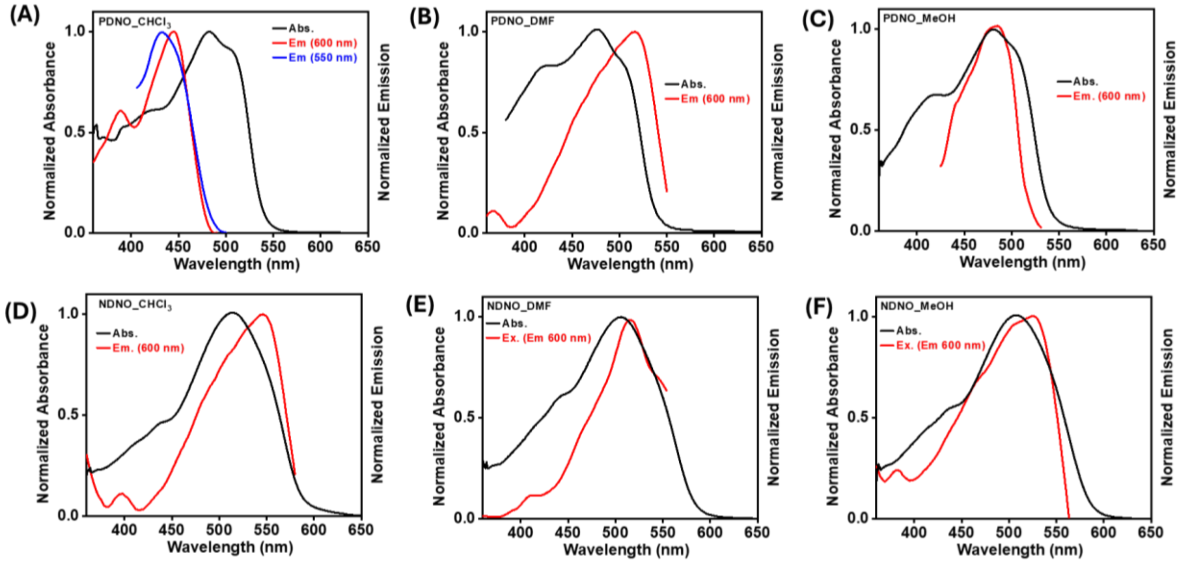

Figure S6. Excitation profiles of **PDNO** and **NDNO** in various solvents.

9. Tables for emission lifetime values of PDNO and NDNO in various solvents.

Table S3. Lifetime values (τ_i's) and relative amplitudes (α_i's) of the emission decays of **PDNO** upon excitation by 450 nm.

Solvent	λ_{mon} (nm)	τ_1(ps)	α_1	τ_2(ns)	α_2	τ_3(ns)	α_3
	500		54		32		14
	520		85		11		4
$CHCl_3$	550	90±10	88	1.2±0.20	8	4.5±0.6	4
	600		90		5		5
	650		94		5		1
	500		87		8	4.0±0.5	3
	520		97	1.3±0.30	2		1
DMF	550	87±10	98		1		1
	600		98		1		1
	650		99		1		<1
	500		75		7	4.1±0.5	18
	520	75±10	93	2.0±0.09	2		5
MeOH	550		94		1		5
	600		95		1		4
	650		96		1		3

Table S4. Lifetime values (τ_i's) and relative amplitudes (α_i's) of the emission decays of **PDNO** upon excitation by 502 nm.

Solvent	λ_{mon} (nm)	τ_1(ps)	α_1	τ_2(ns)	α_2
$CHCl_3$	600	120±10	91	1.2±0.09	9
	650		96		4
DMF	600	100±12	96	1.3±0.12	4
	650		97		3

Table S5. Lifetime values (τ_i's) and relative amplitudes (α_i's) of the emission decays of **NDNO** upon excitation by 450 nm.

Solvent	λ_{mon} (nm)	τ_1(ps)	α_1	τ_2(ns)	α_2	τ_3(ns)	α_3
$CHCl_3$	500	80±10	75	1.0±0.20	14	6.0±0.70	11
	600		90		06		04
	650		95		04		01
DMF	500	83±10	93	1.3±0.12	04	6.0±0.70	3
	600		95		03		2
	650		97		03		<1
MeOH	500	80±10	45	1.2±0.15	07	3.5±0.24	48
	600		90		06		1
	650		92		06		2

Table S6. Lifetime values (τ_i's) and relative amplitudes (α_i's) of the emission decays of **NDNO** upon excitation by 502 nm.

Solvent	λ_{mon} (nm)	τ_1(ps)	α_1	τ_2(ns)	α_2
$CHCl_3$	550	140±20	83	1.5±0.20	17
	600		87		13
	650		89		11
	700		92		8
DMF	550	130±15	69	2.0±0.40	31
	600		96		4
	650		97		3
	700		98		2
MeOH	550	150±20	63	1.7±0.20	37
	600		86		14
	650		87		13
	700		91		9

10. Chirp corrected fs-TA heatmaps of PDNO & NDNO in various solvents

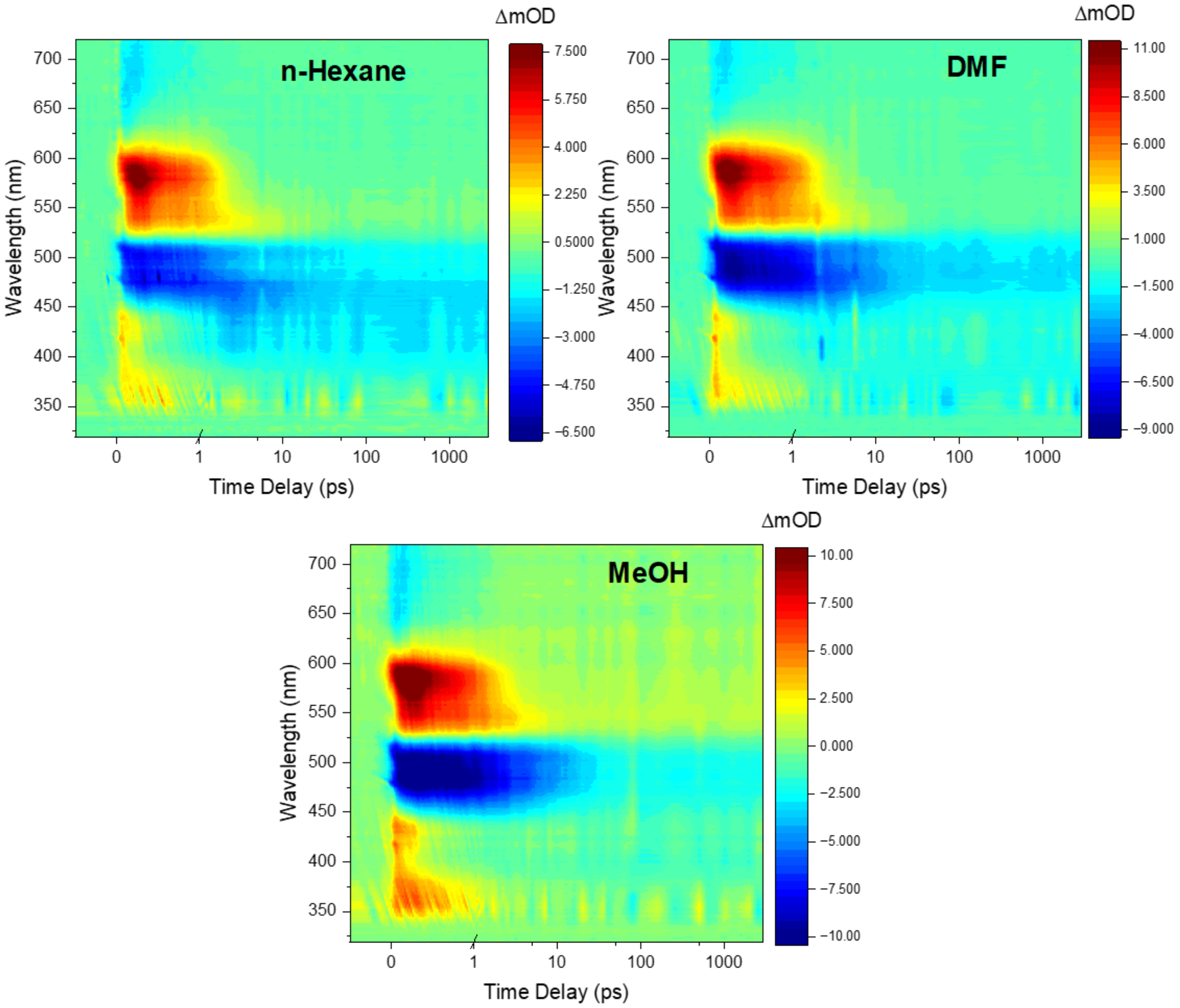

Figure S7. fs-TA heatmaps of **PDNO** in various solvents following photoexcitation at 480 nm. The time delay is linear up to 1 ps and logarithmic from 1 to 2900 ps.

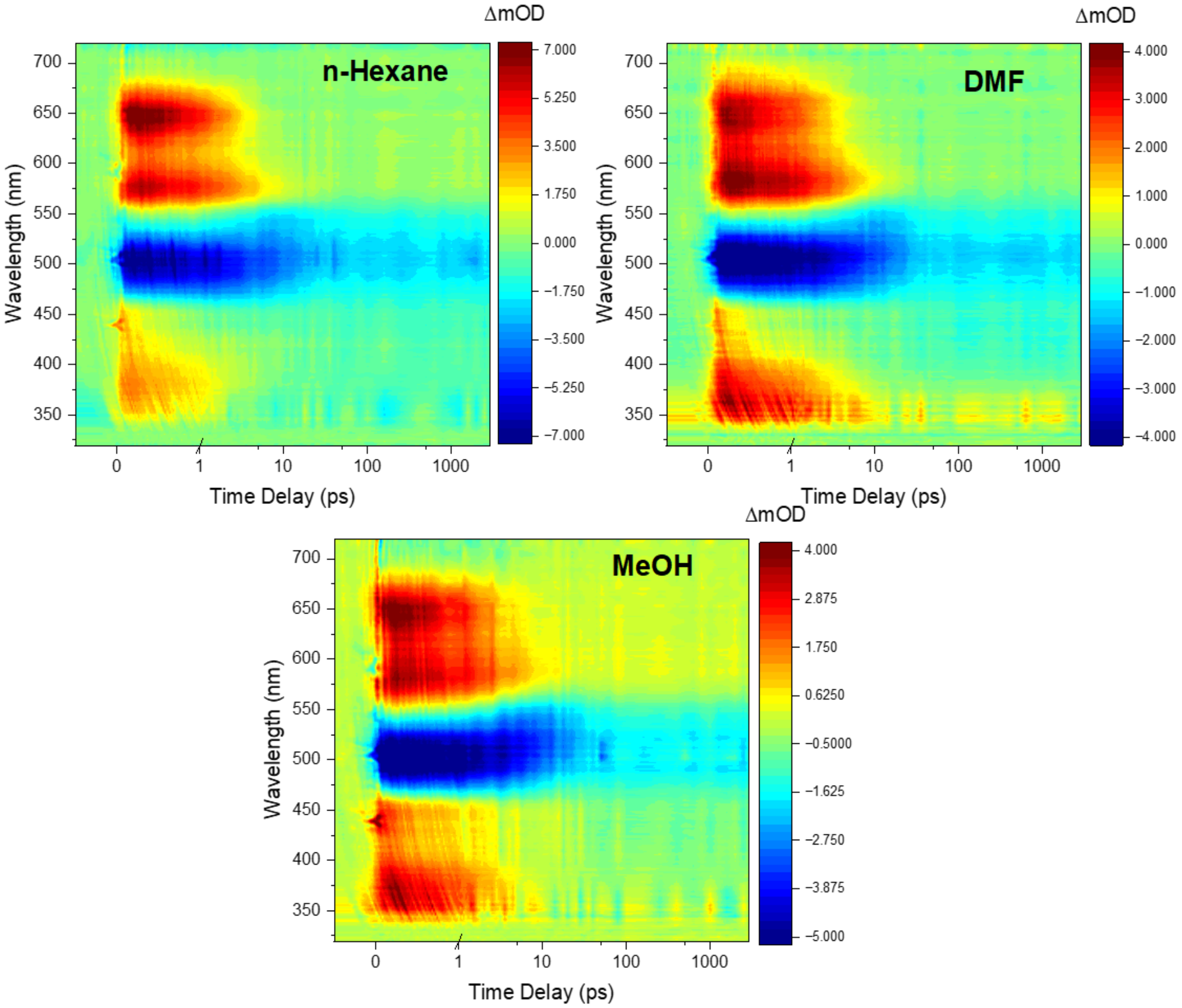

Figure S8. fs-TA heatmaps of **NDNO** in various solvents following photoexcitation at 505 nm. The time delay is linear up to 1 ps and logarithmic from 1 to 2900 ps.

11. EADS of PDNO and NDNO in various solvents.

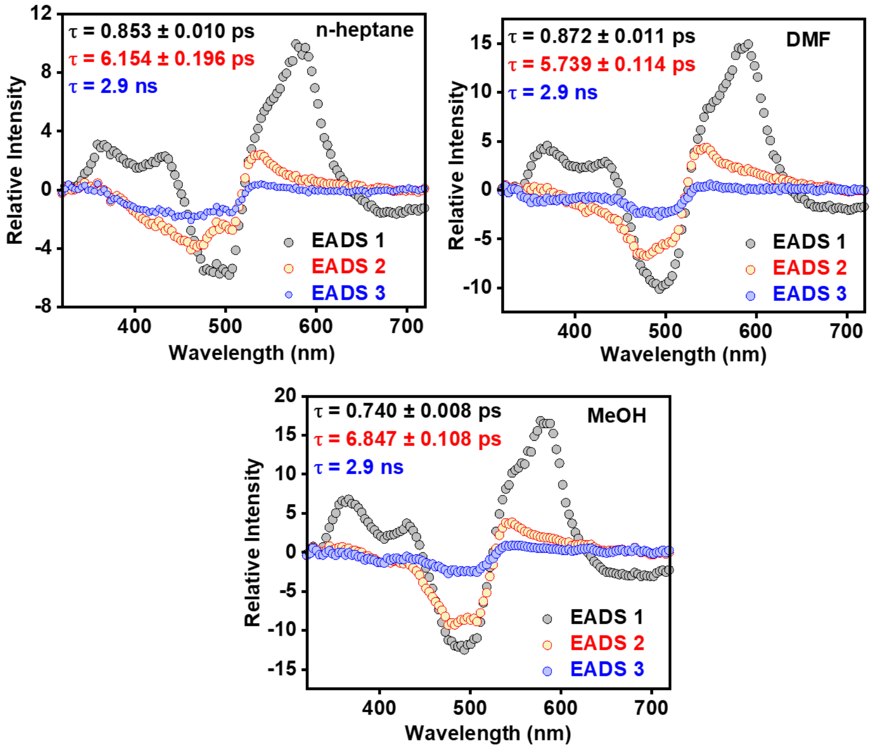

Figure S9. EADS of **PDNO** in various solvents. Corresponding lifetimes are presented along with errors that relate to two standard deviations of the fitting.

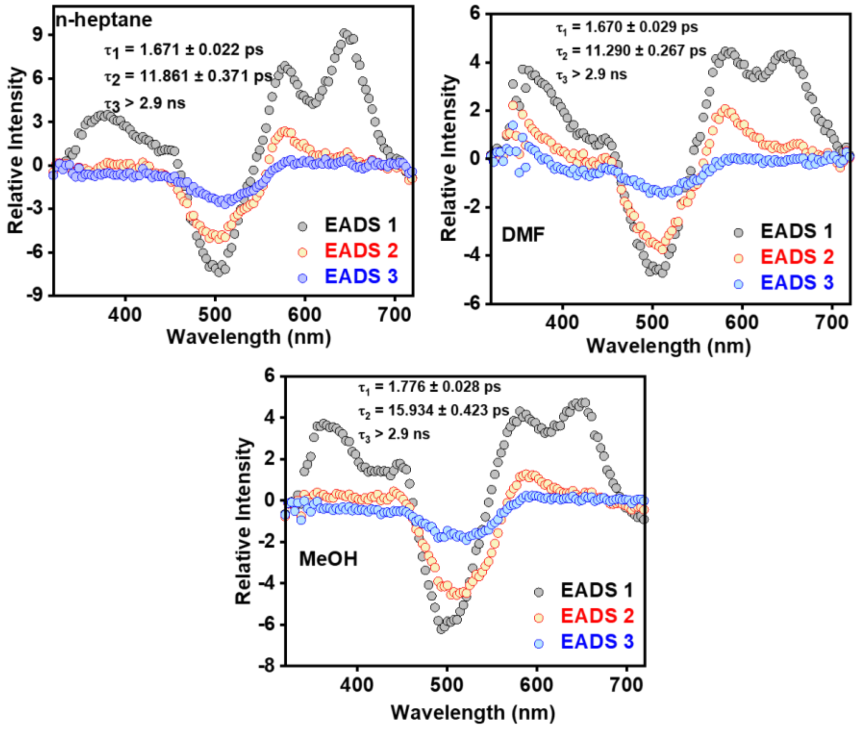

Figure S10. EADS of **NDNO** in various solvents. Corresponding lifetimes are presented along with errors that relate to two standard deviations of the fitting.
